# UBE2C is a Potential Biomarker for Tumorigenesis and Prognosis in Tongue Squamous Cell Carcinoma

**DOI:** 10.3390/diagnostics10090674

**Published:** 2020-09-04

**Authors:** Pei-Feng Liu, Chun-Feng Chen, Chih-Wen Shu, Hui-Min Chang, Cheng-Hsin Lee, Huei-Han Liou, Luo-Ping Ger, Chun-Lin Chen, Bor-Hwang Kang

**Affiliations:** 1 Department of Biomedical Science and Environmental Biology, Kaohsiung Medical University, Kaohsiung 80756, Taiwan; pfliu@kmu.edu.tw (P.-F.L.); angioadsc@gmail.com (C.-H.L.); 2 Department of Medical Research, Kaohsiung Medical University Hospital, Kaohsiung 80756, Taiwan; 3 Center for Cancer Research, Kaohsiung Medical University, Kaohsiung 80756, Taiwan; 4 Institute of Biomedical Sciences, National Sun Yat-sen University, Kaohsiung 80424, Taiwan; cwshu@mail.nsysu.edu.tw; 5 Department of Stomatology, Kaohsiung Veterans General Hospital, Kaohsiung 81362, Taiwan; 261463@gmail.com; 6 School of Dentistry, Kaohsiung Medical University, Kaohsiung 80756, Taiwan; 7 Department of Dental Technology, Shu-Zen Junior College of Medicine and Management, Kaohsiung 821, Taiwan; 8 Institute of Biopharmaceutical Sciences, National Sun Yat-sen University, Kaohsiung 80424, Taiwan; 9 Department of Medical Education and Research, Kaohsiung Veterans General Hospital, Kaohsiung 81362, Taiwan; hmchang790616@vghks.gov.tw (H.-M.C.); slove0726@hotmail.com (H.-H.L.); lpger0329@gmail.com (L.-P.G.); 10 Department of Biological Sciences, National Sun Yat-sen University, Kaohsiung 80424, Taiwan; chunlinchen@mail.nsysu.edu.tw; 11 Department of Otorhinolaryngology-Head and Neck Surgery, Kaohsiung Veterans General Hospital, Kaohsiung 81362, Taiwan; 12 Graduate Institute of Aerospace and Undersea Medicine, National Defense Medical Center, Taipei 114, Taiwan; 13 Department of Pharmacy, Tajen University, Pingtung 907, Taiwan

**Keywords:** UBE2C, tongue squamous cell carcinoma, tumorigenesis, prognosis, biomarker

## Abstract

Ubiquitin-conjugating enzyme 2C (UBE2C) involves in numerous cellular processes and the tumor progression in many cancers. However, its role in oral squamous cell carcinoma (OSCC) is unclear. We aimed to investigate the role and clinical significance of UBE2C in OSCC. The expression levels of UBE2C were examined by immunohistochemistry in 185 buccal mucosa squamous cell carcinomas, 247 tongue squamous cell carcinomas (TSCCs) and 75 lip squamous cell carcinomas. The roles of UBE2C in cell growth, invasion/migration and cancer stemness were also examined in OSCC cells. The expression levels of UBE2C protein were higher in tumor tissues than they were in the corresponding tumor adjacent normal tissues from OSCC patients. Higher UBE2C expression was associated with poor cell differentiation and lymph node invasion in OSCC patients. High UBE2C expression was also correlated with shorter disease-specific survival in TSCC patients having poor cell differentiation, advanced pathological stages, lymph node metastasis as well as receiving radiation therapy. Compared to control cells, OSCC cells in which UBE2C was silenced showed decreased cell proliferation, migration/invasion and colony formation and they exhibited lower expression levels of the following cancer stemness markers—ALDH1/A2, CD44, CD166 and EpCAM. High co-expression levels of UBE2C/CD44, UBE2C/CD166 and UBE2C/EpCAM were associated with poor prognosis in oral cancer patients from The Cancer Genome Atlas database. Our findings indicated that UBE2C might be a potential biomarker for tumorigenesis and prognosis in TSCC.

## 1. Introduction

More than 90% of oral cancers are oral squamous cell carcinoma (OSCC) which is typically observed on the tongue, buccal mucosa and lips [1]. Cigarette smoking, alcohol drinking, betel quid chewing, chronic periodontitis [2] and viral infections [3] are major risk factors contributing to the incidence of OSCC. OSCC is a highly aggressive cancer with frequent local recurrences and lymph node metastases [4]. The overall 5-year survival rate for OSCC patients have not been improved and remained at approximately 50% for several decades [5]. Therefore, how a diagnostic/prognostic assessment of gene expression could be translated into better patient survival. Ultimately, the aim of biomarker tests is probably to identify/stratify patients for certain therapeutic intervention. Thus, identifying reliable diagnostic and prognostic biomarkers for OSCC is urgent.

Human ubiquitin-conjugating enzyme 2C (UBE2C) is a member of the E2 ubiquitin-conjugating enzyme family [6]. Ubiquitin-dependent protein degradation is involved in numerous cell processes, such as cell cycle progression, signal transduction and malignant transformation [7]. UBE2C is required for the destruction of mitotic cyclin and participates in the regulation of cell cycle progression [8]. UBE2C overexpression causes loss of mitotic spindle checkpoint activity and genomic stability [8]. The expression level of UBE2C is very high in various human cancers, such as breast cancer [9], ovarian cancer [10], head and neck squamous cell carcinoma (HNSCC) [11] and melanoma [12], suggesting that UBE2C is associated with tumorigenesis. Moreover, overexpression of UBE2C is correlated with tumor progression [13], so it acts as a potential prognostic/diagnostic biomarker in various types of tumors [11,14,15]. However, the role of UBE2C in OSCC is still unclear. Herein, we found that high expression level of UBE2C was associated with the tumorigenesis, poor clinicopathological outcomes and poor prognosis in OSCC patients. Moreover, UBE2C played roles in cell growth, migration/invasion and cancer stemness in OSCC cells. Furthermore, The Cancer Genome Atlas (TCGA) oral cancer cohorts with co-expression level of high UBE2C/cancer stemness markers had poor prognosis. Our results indicated the potential roles and clinical significance of UBE2C in TSCC.

## 2. Materials and Methods

### 2.1. Patients and Tissue Specimens

All paraffin-embedded tissues of buccal mucosa squamous cell carcinoma (BMSCC, *n* = 185), tongue squamous cell carcinoma (TSCC, *n* = 247) and lip squamous cell carcinoma (LSCC, *n* = 75) were obtained from patients at the Department of Pathology at KVGH between 1990 and 2013. This study was approved by the Institutional Review Board of the Kaohsiung Veterans General Hospital (IRB, approved on: 13-06-14, code number: VGHKS14-CT6-18). Informed consent was obtained from patients before collecting their tissue specimens. Clinicopathological data about TNM stages were determined according to the eight editions of the AJCC Cancer Staging Manual [16]. The survival time of patients was calculated from the date of the primary tumor operation to the date of death or the last follow-up until October 2013.

### 2.2. Tissue Microarray (TMA) Construction

All TMA blocks were constructed with a 1.5 mm diameter and each block consisted of 134 cores, including 43 sets of three (each set of three contained 2 cores from the tumor tissue, 1 core from the corresponding tumor adjacent normal (CTAN) of the same patient) and another 5 cores of normal uvula epithelium. To avoid false positives, noncancerous tissue of patients was excluded. After exclusion, a total of 12 TMA blocks were constructed and subsequently cut into 4-μm paraffin sections.

### 2.3. IHC Analysis and Scoring

Before IHC analysis, paraffin sections were first dewaxed in xylene then rehydrated in a graded alcohol series (30–100%). Then, IHC analysis was performed by using the Novolink Polymer Detection Systems (Leica Biosystem, RE7280-K, Richmond, IL, USA). First, these dewaxed and rehydrated sections were immersed in sodium citrate (pH 6.0) for 10 min at 125 °C in a pressure boiler for antigen retrieval. Then, they were incubated in methanol containing 3% hydrogen peroxide for 30 min to block endogenous peroxidase activity. Afterward, these sections were incubated with an anti-UBE2C monoclonal antibody (dilution 1:500; H00011065-M01, Abnova, Walnut, CA, USA) in 5% BSA/TBS at 4 °C overnight and washed with TBS. The sections were incubated with Poly-HRP-labeled secondary antibody (Novolink™ Polymer) for 30 min, developed with 0.03% diaminobenzidine for 5 min and counterstained with hematoxylin. The IHC staining was scored according intensity and percentage as described in our previous study [17,18]. For the association of UBE2C with patient survival, the ‘high’ and ‘low’ expression level of cytoplasmic UBE2C was defined based on the cutoff point, which was set at the 50th percentile according to the immunoreactive scores of UBE2C.

### 2.4. Cell Culture

Two OSCC cell lines from tongue squamous cell carcinoma (SAS) and gingival squamous cell carcinoma (Ca9-22) were grown in Dulbecco’s modified Eagle’s medium (DMEM, Invitrogen-GIBCO, Carlsbad, CA, USA) containing 10% heat-inactivated fetal bovine serum (Biological Industries, Cromwell, CT, USA), 100 U/mL penicillin (Invitrogen-GIBCO, Carlsbad, CA, USA), 1% MEM nonessential amino acids and 100 µg/mL streptomycin (Invitrogen-GIBCO, Carlsbad, CA, USA) at 37 °C in humidified 5% CO_2_ atmosphere.

### 2.5. siRNA Knockdown and Transient Transfection

UBE2C siRNA pools were synthesized (5′-UCCUUUUUGUGAUUUCUGUTT-3′, Ambion, Austin, TX, USA) and cells were transfected for 48–96 h with 5 nM scrambled siRNA or an siRNA against UBE2C using RNAiMAX (Life Technologies, Carlsbad, CA, USA). The knockdown efficiency was determined by western blot.

### 2.6. Real-Time PCR (RT-PCR)

Total RNA was extracted with TRIzol reagent (Biocompare, South San Francisco, CA, USA). A total of 1 μg of RNA was reverse-transcribed with SuperScriptIII RNase Reverse Transcriptase (Invitrogen-GIBCO, Carlsbad, CA, USA) for cDNA synthesis. Real-time polymerase chain reaction (PCR) was carried out using a StepOnePlus^TM^ system (Applied Biosystems, Foster City, CA, USA) with SYBR Green Master Mix (Applied Biosystems, Foster City, CA, USA). All genes expression levels in cells was normalized with internal control GAPDH gene. The UBE2C primers sequences were shown as follows—the forward—5′-TGATGTCTGGCCATAAAGGGA-3′, the reverse—5′-AGCGAGAGCTTATACCTCAGG-3′.

### 2.7. Western Blot Analysis

Cells were initially washed with phosphate-buffered saline (PBS) and lysed with RIPA buffer (1% NP40, 50 mM Tris Cl pH 7.5, 150 mM NaCl, 0.25% sodium deoxycholate 1% sodium dodecyl sulphate (SDS) and a protease inhibitor cocktail). All proteins were separated by SDS-PAGE and transferred to nitrocellulose membranes. The membranes were incubated with primary anti-UBE2C antibodies (Abnova, Walnut, CA, USA) at 4 °C overnight and then probed with an HRP-labeled secondary antibody (Santa Cruz Biotechnology sc-2004, Santa Cruz, CA, USA). Finally, a LI-COR Odyssey Imaging System (LI-COR Inc, Lincoln, NE, USA) was used to analyze protein expression levels on the probed membrane before incubation with an enhanced chemiluminescence (ECL) reagent (Thermo Fisher Scientific, 32106, Carlsbad, CA, USA).

### 2.8. Cell Viability

Cells were seeded into 96-well flat bottom plates (5 × 10^3^ cells/well) containing 100 µL of medium. Cell viability was determined by Cell Titer-Glo^®^ Luminescent Cell Viability Assay (Promega, Madison, WI, USA). The resultant luminescence signal was measured using a Fluoroskan Ascent FL reader (Thermo Fisher Scientific, Carlsbad, MA, USA).

### 2.9. Colony Formation

1 × 103. cells were seeded into each well of 6-well plates and cultured for 2–3 weeks until colony formation; fresh media was provided every 3 days. The colonies were fixed with paraformaldehyde (3.75% *v*/*v*), stained with crystal violet (0.25% *w*/*v*) and quantified.

### 2.10. Migration and Invasion

For the migration assay, UBE2C-knockdown cells (2 × 10^5^ cells) were cultured for 24 h in dishes fitted with IBIDI Culture-Inserts (35 mm with high culture-insert coating). Afterward, the plastic inserts were removed, wound healing was observed for 7 h and the migration distance was measured. For the invasion assay, UBE2C-knockdown cells were seeded (1.5 × 10^5^ cells/well) into the top chamber of transwell inserts with 8-μm pores (Greiner Bio-One, Monroe, NC, USA) coated with 0.5% Matrigel in 300 μL of DMEM containing 1% Fetal Bovine Serum (FBS). Twenty-four hours after seeding, the cells on the bottom of the inserts were fixed, stained with 0.1% crystal violet and quantified.

### 2.11. Statistical Analysis and TCGA Database

All statistical analyses were performed with SPSS software (version 20.0, SPSS Inc., Chicago, IL, USA). Wilcoxon signed-rank tests were used to compare the protein expression levels between tumor tissues and tumor-adjacent normal tissues. Student’s t-test, one-way ANOVA, Mann-Whitney U test and Kruskal-Wallis one-way ANOVA were used to evaluate the correlation of UNE2C expression levels at different subsites of OSCC with clinicopathologic outcomes. A Cox proportional hazard model and a log-rank test were used to evaluate the contribution of UBE2C expression to disease-specific survival (DSS) in OSCC patients. The association of co-expression level of UBE2C/cancer stemness markers with survival was analyzed using RNA-sequencing transcriptome profiles of 315 oral cancer patients from TCGA database (https://cancergenome.nih.gov). The gene expression levels of UBE2C and cancer stemness markers were dichotomized into ‘high’ and ‘low’ expression according to a receiver operating characteristic (ROC) curve.

## 3. Results

### 3.1. The Clinical Significance of UBE2C in OSCC Patients

**Figure 1:**
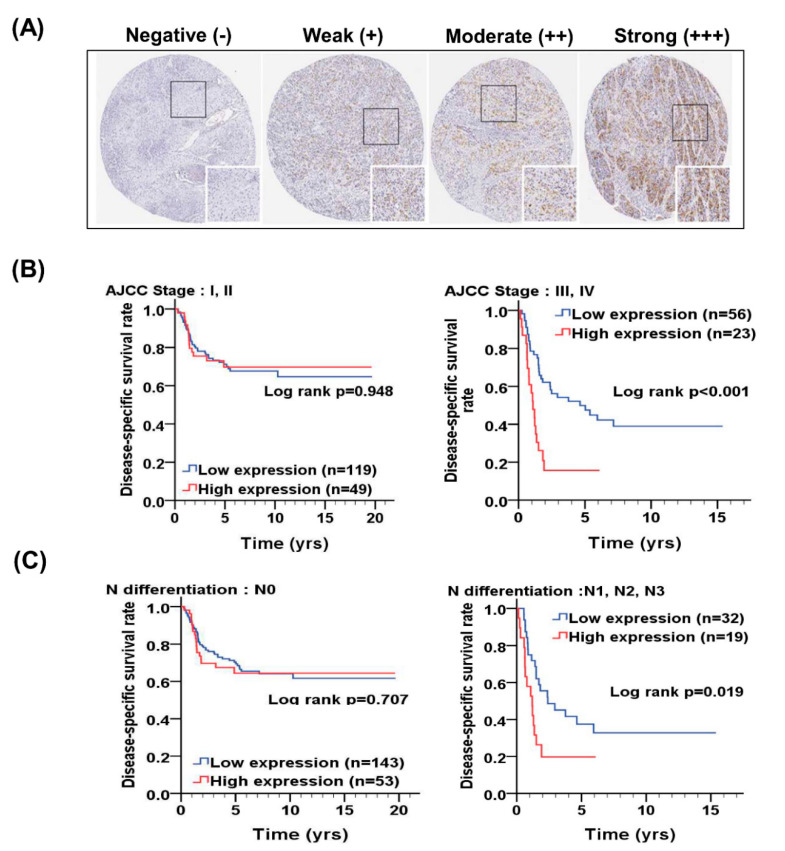
Representative immunohistochemical staining of UBE2C in tumor tissues and compared DSS of OSCC patients according to expression level of UBE2C. (**A**) The expression level of UBE2C was determined using a numerical scale (no signal (−), weak (+), moderate (++) or strong (+++) staining) for scoring intensity. (**B**) The comparison of DSS in OSCC patients with early pathological stage (I + II) and advanced pathological stage (III + IV) according to expression level of UBE2C. (**C**) The comparison of DSS in OSCC patients without lymph node metastasis (N0) and with lymph node metastasis (N1, N2, N3) according to expression level of UBE2C.

To investigate the clinical significance of UBE2C in OSCC patients, the expression level of UBE2C in tissues of OSCC patients was evaluated by scoring of IHC staining according intensity and percentage. For scoring intensity, the expression level of UBE2C in OSCC patients was determined using a numerical scale (−, negative; +, weak; ++, moderate; and +++, strong; Figure 1). After multiplying scores of intensity and percentage in positive cells of each tissues section, we found that the expression level of UBE2C was higher in tumor tissues than it was in CTAN tissues in all subsites of OSCC (*p* < 0.001; Table 1). Moreover, the high expression level of UBE2C was associated with lymph node invasion (*p* = 0.017; Supplementary Table S1) and poor cell differentiation (*p* < 0.001; Supplementary Table S1) in OSCC patients, especially in BMSCC (*p* = 0.002; Supplementary Table S1) and TSCC (*p* = 0.001; Supplementary Table S1). Furthermore, a high expression level of UBE2C was associated with shorter DSS in TSCC patients with poor cell differentiation (*p* = 0.042; Table 2), advanced pathological stage (*p* = 0.001; Table 2), lymph node metastasis (*p* = 0.041; Table 2) and postoperative radiation therapy (*p* = 0.002; Table 2). By log rank test, we found that OSCC patients with high UBE2C level had a significantly shorter DSS, especially for those with advanced pathological stage (Figure 1B, *p* < 0.001) and lymph node metastasis (Figure 1C, *p* = 0.019). Taken together, our results indicated that UBE2C expression was significantly associated with tumorigenesis, clinicopathological outcomes and prognosis in OSCC patients, especially in TSCC patients.

### 3.2. The role of UBE2C in the Growth of OSCC Cells

**Figure 2:**
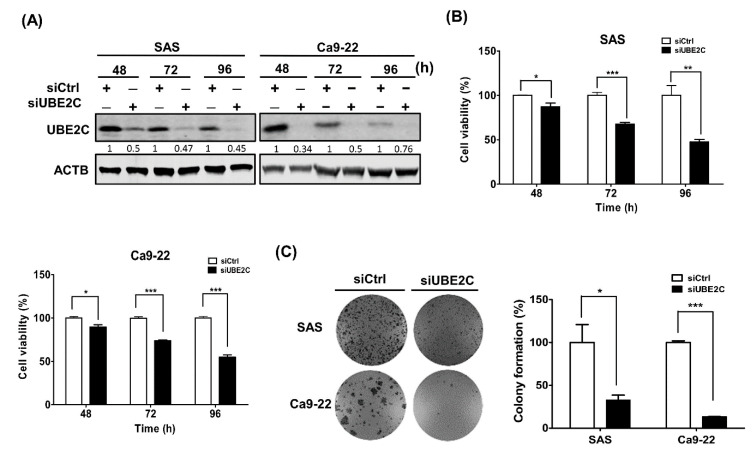
Cell viability and colony formation in UBE2C-knockdown SAS and Ca9-22 cells. (**A**) UBE2C knockdown efficiency of cells transfected with scrambled siRNA (5 nM, siCtrl) or siRNAs against UBE2C (5 nM, siUBE2C) for 48, 72 and 96 h was analyzed by western blot analysis. (**B**) The viability of cells transfected with scrambled siRNA (5 nM, siCtrl) or siRNAs against UBE2C (5 nM, siUBE2C) for 48 h was measured by Cell Titer Glo. (**C**) The colony formation of cells transfected with scrambled s siRNA (5 nM, siCtrl) or siRNAs against UBE2C (5 nM, siUBE2C) was monitored for 1–3 weeks. The colonies of UBE2C-knockdown cells are shown in the left panel and their quantification is shown in the right panel. The quantitative results are expressed as the mean ± SD from three independent experiments. * *p* < 0.05, ** *p* < 0.01 and *** *p* < 0.001 vs. nontargeting control siRNA (siCtrl).

Our results indicated that UBE2C expression was associated with tumorigenesis and prognosis in TSCC patients. To further investigate the role of UBE2C in the tumorigenesis of TSCC, UBE2C was knocked down in two OSCC cell lines, SAS and Ca9-22 cells, for 48, 72 and 96 h and the knockdown efficiency was verified by western blot (Figure 2A). Moreover, we found that cell viability (Figure 2B) and colony formation (Figure 2C) were significantly decreased in UBE2C-knockdown OSCC cells compared to control cells. These results suggested that UBE2C might be involved in the growth of OSCC cells.

### 3.3. The Role of UBE2C in Invasion and Migration of OSCC Cells

**Figure 3:**
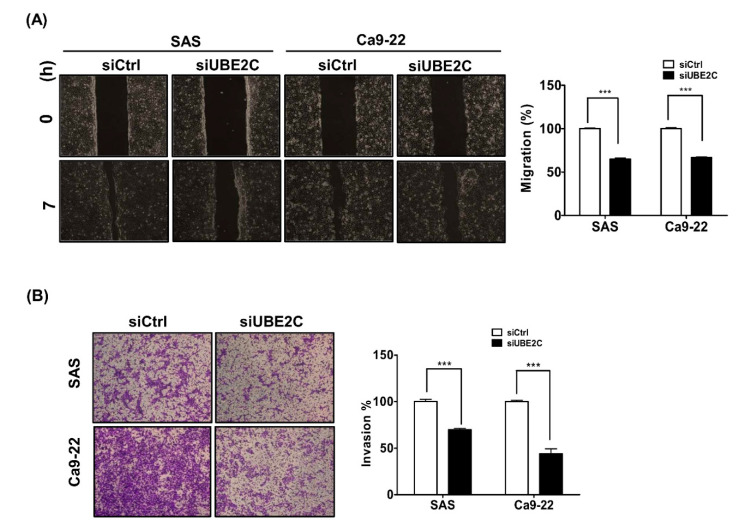
Cell migration and invasion in UBE2C-knockdown SAS and Ca9-22 cells. (**A**) Cells were transfected with scrambled siRNA (5 nM, siCtrl) or siRNAs against UBE2C (5 nM, siUBE2C) for 7 h and then their migration was assessed by wound–healing assay. (**B**) The invasion of cells transfected with scrambled siRNA (5 nM, siCtrl) or siRNAs against UBE2C (5 nM, siUBE2C) for 24 h was measured by transwell invasion assay. The quantitative results are expressed as the mean ± SD from three independent experiments. * *p* < 0.05, ** *p* < 0.01, *** *p* < 0.001 vs. nontargeting control siRNA (siCtrl).

To further investigate the possible role of UBE2C in the metastasis of TSCC, UBE2C was knocked down in SAS and Ca9-22 cells by transfecting with an siRNA against UBE2C for 48 h and their invasion and migration abilities were subsequently evaluated. The results showed that the migration of UBE2C-knockdown cells was significantly suppressed by 65% and 67% in SAS and Ca9-22 cells compared to that of control cells (Figure 3A), respectively. Moreover, UBE2C-knockdown SAS and Ca9-22 cells also showed decreased invasion by 70% and 44%, respectively (Figure 3B). The results indicated that UBE2C might be involved in OSCC metastasis.

### 3.4. The Role of UBE2C in Cancer Stemness of OSCC Cells

**Figure 4:**
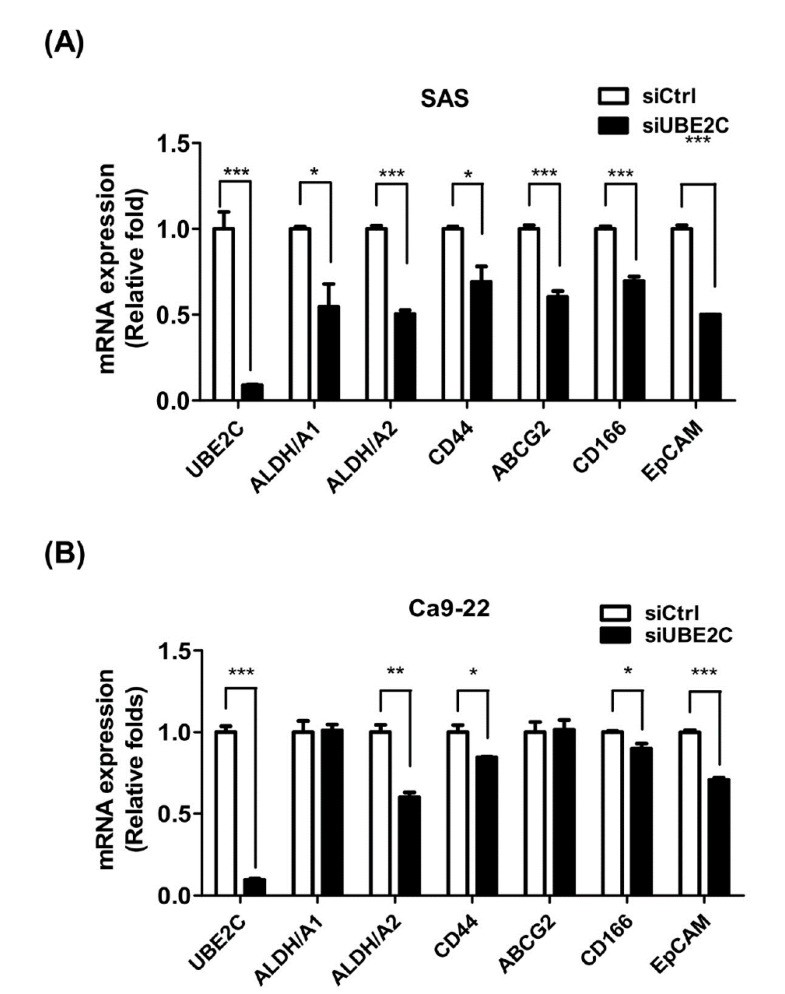
Expression of cancer stemness markers in UBE2C-knockdown OSCC cells. (**A**) SAS cells. (**B**) Ca9-22 cells. The expression levels of the ALDH1/A1, ALDH1/A2, CD44, ABCG2, CD166 and EpCAM in cells transfected with scrambled s siRNA (5 nM, siCtrl) or siRNAs against UBE2C (5 nM, siUBE2C) were detected by real time polymerase chain reaction (RT-PCR) and GAPDH was used as an internal control. The quantitative results are expressed as the mean ± SD from three independent experiments. * *p* < 0.05, ** *p* < 0.01 and *** *p* < 0.001 vs. nontargeting control siRNA (siCtrl).

UBE2C plays roles in some cancer stemness properties, such as drug resistance [19,20]. To investigate whether UBE2C is also involved in cancer stemness in TSCC, the expression of several cancer stemness markers was evaluated by RT-PCR. The expression levels of aldehyde dehydrogenase A2 (ALDH1A2), CD44, CD166 and epithelial cell adhesion molecule (EpCAM) were significantly decreased in UBE2C-knockdown SAS (Figure 4A) and Ca9-22 cells (Figure 4B). Moreover, high coexpression of UBE2C/CD44, UBE2C/CD166 and UBE2C/EpCAM genes (Table 3) was associated with overall survival and recurrence in oral cancer patients including TSCC patients from TCGA database, indicating that UBE2C might be involved in controlling cancer stemness of OSCC.

## 4. Discussion

UBE2C is involved in tumor progression and is considered a potential cancer biomarker [21,22]. However, its role has not been reported in OSCC, especially for TSCC. In the present study, we first indicated that (1) the expression level of UBE2C was higher in tumor tissues of OSCC patients than it was in CTAN tissues; (2) the high expression level of UBE2C in tumor tissues was associated with poor cell differentiation and lymph node invasion in OSCC patients, especially in BMSCC and TSCC patients; (3) the high expression level of UBE2C in tumor tissues was associated with shorter DSS in TSCC patients with poor cell differentiation, advanced pathological stage, lymph node metastasis and postoperative radiation therapy; (4) UBE2C was involved in cell proliferation, migration, invasion, colony formation and cancer stemness of OSCC cells; and (5) the high co-expression levels of UBE2C and cancer stemness markers such as CD44, CD166 and EpCAM were associated with poor prognosis in oral cancer patients including TSCC patients from TCGA database. These findings suggested the potential clinical significance and roles of UBE2C in OSCC.

Overexpression of UBE2C is associated with tumorigenesis and tumor progression in various types of cancer. For example, UBE2C is involved in the tumorigenesis of colorectal cancer [23] and non–small cell lung cancer (NSCLC) [24]. UBE2C is highly expressed in breast microcalcification lesions [25] and its overexpression is correlated with relapse in early HR^+^/HER2^−^ breast cancer patients [9]. UBE2C expression is elevated and predicts poor prognosis in hepatocellular carcinoma, esophageal squamous cell carcinoma and intestinal-type gastric cancer [14,26,27]. UBE2C promotes the progression of HNSCC [11] and nasopharyngeal carcinoma progression [28]. High expression of UBE2C is associated with the tumor progression and unfavorable outcome in patients with malignant glioma [29]. UBE2C expression was positively correlated with unfavorable overall survival in ovarian and bladder cancers [30,31]. High expression levels of UBE2C were correlated with high rates of tumor recurrence in meningiomas [32]. Similarly, our results showed that UBE2C was highly expressed in the tumor tissues of OSCC patients and that a high expression level of UBE2C was associated with shorter DSS in TSCC patients having poor cell differentiation, lymph node metastasis and postoperative radiation therapy, indicating that UBE2C might be a potential diagnostic and prognostic biomarker in OSCC patients, especially in TSCC patients with certain clinicopathological outcomes.

UBE2C is a key regulator of cell cycle progression in various types of cancers, such as NSCLC, gastric cancer and breast cancer [33]. Silencing UBE2C arrests cell cycle progression at the G_1_/S phases, inhibits cell proliferation in pancreatic ductal adenocarcinoma [34] and blocks the G_2_/M transition in melanoma [12]. Moreover, the downregulation of UBE2C suppresses cell proliferation in osteosarcoma by decreasing the expression of the cell cycle-related protein Ki-67 [35]. UBE2C overexpression accelerates the proliferation of colon cancer by alternating the cell cycle profile [36]. On the other hand, knockdown of UBE2C arrests G_2_/M phase of cell cycle and enhances cell apoptosis through induction of Bax/p53 and downregulation of Bcl-2 [37]. Our results indicated that silencing UBE2C decreased the viability of OSCC cells (Figure 2) but further verification is needed to determine whether UBE2C is involved in cell proliferation or apoptosis.

UBE2C also modulates migration and invasion in various types of cancers, such as hepatocellular carcinoma [13], NSCLC [7,24], HNSCC [11] and pancreatic ductal adenocarcinoma [34]. The expression of UBE2C is correlated with lymphatic metastasis and serosa invasion [15]. Moreover, UBE2C induces Wnt/β-catenin and PI3K/Akt signaling to regulate phosphorylation levels of Aurora-A for epithelial–mesenchymal transition (EMT) in gastric adenocarcinoma [38]. UBE2C promotes EMT via p53 and p21 in endometrial cancer [39]. Our results indicated that UBE2C was involved in cell invasion and migration in OSCC cells (Figure 3); higher UBE2C expression was associated with lymph node metastasis in OSCC patients (Supplementary Table S1) and with poor DSS in TSCC patients having lymph node metastasis (Table 3). These results indicated that UBE2C was associated with TSCC metastasis but their molecular mechanisms regarding metastasis also need to be further investigated.

Knockdown of UBE2C sensitizes epirubicin- and docetaxel-resistant breast cancer cells to chemotherapeutic agents [40]. Moreover, UBE2C induces cisplatin resistance in NSCLC cells [41]. These findings suggested that UBE2C plays an important role in drug resistance. However, the role of UBE2C in the drug resistance of OSCC is not clear. Cancer stem cells are responsible for drug resistance and to contribute to incomplete therapeutic responses of tumors [20]. Cancer stemness markers such as ALDH1/A2, CD44, CD166 and EpCAM are associated with drug resistance in cancers [42]. Among them, CD44 is involved in drug resistance in oral cancer [43]. Our results indicated that knockdown of UBE2C decreased the expression levels of these cancer stemness markers including ALDH1A2, CD44, CD166 and EpCAM in both Ca9-22 and SAS cells (Figure 4). However, reduced expression levels of ADLH/A1 and ABCG2 stemness markers were only found in UBE2C-knockdown Ca9-22 cells but not in UBE2C-knockdown SAS cells, which indicating that UBE2C might play a more important role in cancer stemness in tongue carcinoma. Moreover, high co-expression of UBE2C/CD44, UBE2C/CD166 and UBE2C/EpCAM was associated with recurrence in oral cancer patients including TSCC patients (Table 3). These results indicated that UBE2C might be involved in stemness–related properties of drug resistance in TSCC.

Indeed, UBE2C expression is associated in worse survival in several cancer types except oral cancer (https://www.proteinatlas.org/ENSG00000175063-UBE2C), suggesting low discrimination of UBE2C expression between different forms of cancers. Nevertheless, we further found that high co-expression of UBE2C and cancer stemness markers, including CD44, D166 and EpCAM, have even worse poor prognosis in TSCC patients, which provided potential diagnostic and prognostic markers in oral cancer patients. Although our study indicated that UBE2C played the role in cancer stemness through regulating several cancer stemness markers including ALDH1A2, CD44, CD166 and EpCAM in oral cancer, their detailed molecular mechanisms for cancer stemness are still largely unresolved, which require further work to elucidate. More studies will be focused on investigating how UBE2C regulating these cancer markers including ALDH1A2, CD44, CD166 and EpCAM and the relationship between these cancer markers for cancer stemness in vitro. The regulation pathway of UBE2C-modulating cancer stemness will be further validated in oral cancer patient. Moreover, we found that UBE2C expression was associated with worse survival in TSCC patients. To further verify the clinical significance of UBE2C in tumor prognosis of oral cancer, the data from more independent cohorts or using external databases, such as The International Cancer Genome Consortium (ICGC) and the Gene Expression Omnibus (GEO), which will be applied for further analysis.

## 5. Conclusions

In conclusion, our study reported that UBE2C expression was associated with cell growth, invasion/migration and cancer stemness in TSCC. Moreover, a high level of UBE2C expression was associated with tumorigenesis, poor cell differentiation and lymph node invasion in OSCC patients and with poor DSS in TSCC patients having poor cell differentiation, advanced pathological stages and lymph node metastasis and receiving radiation therapy. These new findings indicated that UBE2C may be a potential diagnostic and prognostic biomarker for TSCC patients with certain clinicopathological outcomes.

## Figures and Tables

**Table 1:** Comparison of UBE2C expression between corresponding tumor adjacent normal tissues and tumor tissues in three primary subsites of OSCC patients.

Variables	No.	Tumor Adjacent Normal		Tumor		Z	*p*-Value *
		Mean ± SD	Median	Mean ± SD	Median		
Buccal mucosal SCC	136	0.57 ± 0.92	0.00	2.56 ± 1.33	3.00	9.267	**<0.001**
Tongue SCC	188	0.08 ± 0.34	0.00	1.69 ± 1.38	1.50	10.162	**<0.001**
Lip SCC	63	0.38 ± 0.85	0.00	1.56 ± 1.12	2.00	5.177	**<0.001**
Total: Oral SCC	387	0.30 ± 0.72	0.00	1.97 ± 1.39	2.00	14.717	**<0.001**

Abbreviations: SCC, squamous cell carcinoma; SD, standard deviation. * *p*-values were estimated by Wilcoxon signed-rank test. Bold values denote statistically significant.

**Table 2:** Association of UBE2C expression with disease-specific survival stratified by clinicopathologic factors in three primary subsites of OSCC patients.

		Buccal Mucosal SCC (*n* = 185)			Tongue SCC (*n* = 247)			Lip SCC (*n* = 75)		
Variable	UBE2C	No. (%)	AHR (95% CI)	*p* Value ^†^	No. (%)	AHR (95% CI)	*p* Value ^†^	No. (%)	AHR (95% CI)	*p* Value ^†^
Sex										
Female	Low	1 (25.0)	1.00		17 (58.6)	1.00		4 (57.1)	1.00	
	High	3 (75.0)	Incalculable		12 (41.4)	0.45 (0.08–2.59)	0.370^a^	3 (42.9)	Incalculable	
Male	Low	72 (39.8)	1.00		158 (72.5)	1.00		56 (82.4)	1.00	
	High	109 (60.2)	1.08 (0.67–1.74)	0.751 ^a^	60 (27.5)	1.86 (1.20–2.89)	**0.006 ^a^**	12 (17.6)	2.17 (0.57–8.24)	0.254 ^a^
Age, yrs										
≤5 0	Low	36 (43.4)	1.00		92 (71.9)	1.00		14 (82.4)	1.00	
	High	47 (56.6)	0.76 (0.37–1.54)	0.439 ^a^	36 (28.1)	1.50 (0.86–2.61)	0.156 ^a^	3 (17.6)	1.64 (0.14–18.90)	0.693 ^a^
>50	Low	37 (36.3)	1.00		83 (69.7)	1.00		46 (79.3)	1.00	
	High	65 (63.7)	1.54 (0.79–3.01)	0.207 ^a^	36 (30.3)	1.62 (0.84–3.14)	0.149 ^a^	12 (20.7)	2.06 (0.38–11.15)	0.403 ^a^
Cell differentiation										
Well	Low	26 (52.0)	1.00		22 (81.5)	1.00		29 (82.9)	1.00	
	High	24 (48.0)	0.54 (0.18–1.67)	0.287 ^b^	5 (18.5)	1.48 (0.15–14.40)	0.738 ^b^	6 (17.1)	Incalculable	
Moderate, poor	Low	47 (34.8)	1.00		153 (69.5)	1.00		31 (77.5)	1.00	
	High	88 (65.2)	1.27 (0.74–2.17)	0.387 ^b^	67 (30.5)	1.57 (1.02–2.41)	**0.042 ^b^**	9 (22.5)	0.59 (0.07–4.86)	0.621 ^b^
AJCC pathological stage										
I, II	Low	48 (42.1)	1.00		119 (70.8)	1.00		47 (79.7)	1.00	
	High	66 (57.9)	1.18 (0.56–2.49)	0.664 ^c^	49 (29.2)	0.95 (0.51–1.75)	0.859 ^c^	12 (20.3)	0.88 (0.10–7.98)	0.907 ^c^
III, IV	Low	25 (35.2)	1.00		56 (70.9)	1.00		13 (81.3)	1.00	
	High	46 (64.8)	1.06 (0.58–1.95)	0.842 ^c^	23 (29.1)	2.67 (1.46–4.86)	**0.001 ^c^**	3 (18.8)	4.66 (0.55–39.46)	0.158 ^c^
T classification										
T1, T2	Low	57 (40.7)	1.00		134 (69.1)	1.00		49 (79.0)	1.00	
	High	83 (59.3)	1.23 (0.68–2.22)	0.489 ^d^	60 (30.9)	1.45 (0.88–2.40)	0.144 ^d^	13 (21.0)	0.87 (0.17–4.50)	0.870 ^d^
T3, T4	Low	16 (35.6)	1.00		41 (77.4)	1.00		11 (84.6)	1.00	
	High	29 (64.4)	0.99 (0.45–2.18)	0.982 ^d^	12 (22.6)	1.62 (0.71–3.71)	0.251 ^d^	2 (15.4)	3.08 (0.13–74.18)	0.488 ^d^
N classification										
N0	Low	57 (41.0)	1.00		143 (73.0)	1.00		56 (80.0)	1.00	
	High	82 (59.0)	1.03 (0.55–1.93)	0.938 ^e^	53 (27.0)	1.21 (0.69–2.12)	0.496 ^e^	14 (20.0)	1.77 (0.36–8.84)	0.486^e^
N1, N2	Low	16 (34.8)	1.00		32 (62.7)	1.00		4 (80.0)	1.00	
	High	30 (65.2)	1.29 (0.62–2.66)	0.493 ^e^	19 (37.3)	2.06 (1.03–4.14)	**0.041 ^e^**	1 (20.0)	1.41 (0.09–23.57)	0.809 ^e^
Postoperative RT										
No	Low	55 (41.7)	1.00		125 (69.4)	1.00		57 (79.2)	1.00	
	High	77 (58.3)	1.40 (0.75–2.61)	0.290 ^a^	55 (30.6)	1.18 (0.69–2.02)	0.544 ^a^	15 (20.8)	1.71 (0.45–6.57)	0.432 ^a^
Yes	Low	18 (34.0)	1.00		50 (74.6)	1.00		3 (100.0)	1.00	
	High	35 (66.0)	0.79 (0.38–1.63)	0.522 ^a^	17 (25.4)	3.01 (1.49–6.09)	**0.002 ^a^**	0 (0.0)	Incalculable	

Abbreviations: CHR, crude hazard ratio; CI, confidence interval; AHR, adjusted hazard ratio; AJCC, American Joint Committee on Cancer; RT, radiotherapy. † *p* values were estimated by multivariate Cox’s regression. ^a^ Adjusted for cell differentiation (moderate + poor vs. well) and AJCC pathological stage (stage III + IV vs. stage I + II). ^b^ Adjusted for AJCC pathological stage (stage III + IV vs. stage I + II). ^c^ Adjusted for cell differentiation (moderate + poor vs. well). ^d^ Adjusted for cell differentiation (moderate + poor vs. well) and N classification (N1, N2 vs. N0). ^e^ Adjusted for cell differentiation (moderate+poor vs. well) and T classification (T3, T4 vs. T1, T2). Bold values denote statistically significant.

**Table 3:** Association of co-expression of UBE2C and cancer stemness markers with survival in oral cancer patients from TCGA database.

Variable	No. (%)	CHR (95% CI)	*p* Value *	AHR (95% CI)	*p* Value ^†^
**Overall survival**					
UBE2C (L) CD44(L)	38 (14.4)	1.00		1.00	
UBE2C (H) CD44(L)	141 (53.4)	1.30 (0.90–1.88)	0.166	2.91 (1.39–6.08)	**0.004**
UBE2C (L) CD44(H)	17 (6.4)	0.74 (0.34–1.59)	0.435	1.91 (0.69–5.30)	0.212
UBE2C (H) CD44(H)	68 (25.8)	1.38 (0.92–2.07)	0.115	3.29 (1.51–7.16)	**0.003**
UBE2C (L) ALCAM(L)	51 (19.3)	1.00		1.00	
UBE2C (H) ALCAM(L)	171 (64.8)	1.12 (0.76–1.65)	0.562	2.24 (1.23–4.07)	**0.008**
UBE2C (L) ALCAM(H)	4 (1.5)	0.83 (0.21–3.37)	0.795	1.81 (0.41–8.07)	0.438
UBE2C (H) ALCAM(H)	38 (14.4)	2.05 (1.32–3.18)	**0.001**	3.91 (1.99–7.68)	**<0.001**
UBE2C (L) EPCAM(L)	51 (19.3)	1.00		1.00	
UBE2C (H) EPCAM(L)	141 (53.4)	1.21 (0.84–1.75)	0.308	2.42 (1.31–4.50)	0.005
UBE2C (L) EPCAM(H)	4 (1.5)	0.90 (0.28–2.91)	0.862	1.93 (0.53–6.98)	0.319
UBE2C (H) EPCAM(H)	68 (25.8)	2.50 (1.01–2.24)	**0.045**	2.99 (1.55–5.78)	**0.001**
**Disease-free survival**					
UBE2C (L) CD44(L)	37 (16.4)	1.00		1.00	
UBE2C (H) CD44(L)	10 (4.4)	2.15 (0.77–5.95)	0.143	3.90 (1.04–14.57)	**0.043**
UBE2C (L) CD44(H)	149 (65.9)	0.82 (0.47–1.40)	0.461	1.75 (0.69–4.48)	0.242
UBE2C (H) CD44(H)	30 (13.3)	1.89 (1.00–3.60)	**0.051**	3.21 (1.13–9.12)	**0.029**
UBE2C (L) ALCAM(L)	130 (57.5)	1.00		1.00	
UBE2C (H) ALCAM(L)	18 (8.0)	0.98 (0.35–2.70)	0.961	1.15 (0.40–3.30)	0.792
UBE2C (L) ALCAM(H)	56 (24.8)	0.80 (0.42–1.52)	0.492	0.99 (0.50–1.96)	0.977
UBE2C (H) ALCAM(H)	22 (9.7)	2.84 (1.50–5.40)	**0.001**	2.87 (1.45–5.67)	**0.002**
UBE2C (L) EPCAM(L)	158 (69.9)	1.00		1.00	
UBE2C (H) EPCAM(L)	32 (14.2)	1.25 (0.61–2.56)	0.537	1.42 (0.68–2.97)	0.354
UBE2C (L) EPCAM(H)	28 (12.4)	0.78 (0.33–1.82)	0.565	0.93 (0.39–2.23)	0.878
UBE2C (H) EPCAM(H)	8 (3.5)	4.99 (2.24–11.12)	**<0.001**	5.24 (2.30–11.95)	**<0.001**

Abbreviations: OSCC, Oral squamous cell carcinoma; CHR, crude hazard ratio; CI, confidence interval; AHR, adjusted hazard ratio; AJCC, American Joint Committee on Cancer; RT, radiotherapy. * *p* values were estimated by Cox’s regression. ^†^ *p* values were adjusted for cell differentiation (moderate + poor vs. well) and AJCC pathological stage (stage III + IV vs. stage I + II) by multivariate Cox’s regression. Bold values denote statistically significant.

## Supplementary Materials

The following are available online at https://www.mdpi.com/2075-4418/10/9/674/s1, Table S1. Association of UBE2C expression with clinicopathologic outcomes in three primary subsites of OSCC patients.
