# Supplementary material for: UBE2C is a Potential Biomarker for Tumorigenesis and Prognosis in Tongue Squamous Cell Carcinoma
Source: Diagnostics (Basel). 2020 Sep 4;10(9):674. doi: 10.3390/diagnostics10090674 (PMC7555092; doi:10.3390/diagnostics10090674)
Supplement: Supplementary file 1 [file diagnostics-10-00674-s001.pdf]

Supplementary Table 1. Association of UBE2C expression with clinicopathologic outcomes in three primary subsites of OSCC patients

Variable	Buccal mucosal SCC (n=185)				Tongue SCC (n=247)				Lip SCC (n=75)				Oral SCC (n=507)			
	%	Mean±SD	Median	*p*-value	%	Mean±SD	Median	*p*-value	%	Mean±SD	Median	*p*-value	%	Mean±SD	Median	*p*-value
Sex																
Female	2.2	3.50 ± 1.29	3.50	0.141*	11.7	1.93 ± 1.33	2.00	0.123*	9.3	2.00 ± 1.15	2.00	0.165*	7.9	2.10 ± 1.35	2.00	0.346*
Male	97.8	2.51 ± 1.32	3.00		88.3	1.51 ± 1.37	1.00		90.7	1.38 ± 1.11	1.00		92.1	1.88 ± 1.41	2.00	
Age, y																
≦50	44.9	2.49 ± 1.37	3.00	0.704*	51.8	1.58 ± 1.38	1.00	0.855*	22.7	1.12 ± 1.17	1.00	0.178*	45.0	1.88 ± 1.44	2.00	0.747*
＞50	55.1	2.57 ± 1.29	3.00		48.2	1.55 ± 1.36	1.00		77.3	1.53 ± 1.10	1.00		55.0	1.92 ± 1.37	2.00	
Cell differentiation																
Well	27.0	2.06 ± 1.32[cd]	2.00	**0.002‡**	10.9	1.07 ± 1.14[e]	1.00	**0.001‡**	46.7	1.37 ± 1.00	1.00	0.867‡	22.1	1.61 ± 1.25[g]	1.00	**<0.001‡**
Moderate	67.6	2.66 ± 1.25[c]	3.00		82.2	1.54 ± 1.35[f]	1.00		46.7	1.51 ± 1.20	2.00		71.6	1.92 ± 1.41[h]	2.00	
Poor	5.4	3.40 ± 1.58[d]	3.00		6.9	2.65 ± 1.37[ef]	3.00		6.7	1.40 ± 1.52	1.00		6.3	2.69 ± 1.55[gh]	3.00	
AJCC pathological stage																
I, II	61.6	2.46 ± 1.26	3.00	0.305*	68.0	1.57 ± 1.30	1.50	0.885*	78.7	1.46 ± 1.15	1.00	0.795*	67.3	1.85 ± 1.33	2.00	0.368§
III, IV	38.4	2.66 ± 1.42	3.00		32.0	1.54 ± 1.52	1.00		21.3	1.38 ± 1.02	1.00		32.7	2.01 ± 1.54	2.00	
T classification																
T1, T2	75.7	2.54 ± 1.31	3.00	0.992*	78.5	1.64 ± 1.37	2.00	0.073*	82.7	1.45 ± 1.15	1.00	0.846*	78.1	1.93 ± 1.39	2.00	0.365*
T3, T4	24.3	2.53 ± 1.38	3.00		21.5	1.26 ± 1.32	1.00		17.3	1.38 ± 0.96	1.00		21.9	1.79 ± 1.43	1.00	
N classification																
N0	75.1	2.44 ± 1.26	3.00	0.085*	79.4	1.50 ± 1.29	1.00	0.340§	93.3	1.44 ± 1.12	1.00	0.935*	79.9	1.81 ± 1.33	2.00	**0.017***
N1, N2	24.9	2.83 ± 1.48	3.00		20.6	1.80 ± 1.64	1.00		6.7	1.40 ± 1.14	1.00		20.1	2.25 ± 1.63	2.00	

Abbreviations: SCC, squamous cell carcinoma; AJCC, American Joint Committee on Cancer.

* *p values were estimated by student's t-test.*

† *p values were estimated by Kruskal-Wallis one-way ANOVA test.*

‡ *p values were estimated by one-way ANOVA test.*

§ *p values were estimated by Mann–Whitney U test.*

[a]p<0.001; [b]p<0.001; [c]p=0.023; [d]p=0.012; [e]p=0.001; [f]p=0.005; [g]p=0.001; [h]p=0.011.

Bold values denote statistically significant
